# Primary cold atmospheric plasma combined with low dose cisplatin as a possible adjuvant combination therapy for HNSCC cells—an in-vitro study

**DOI:** 10.1186/s13005-022-00322-5

**Published:** 2022-06-29

**Authors:** Teresa F. Brunner, Florian A. Probst, Matthias Troeltzsch, Sabina Schwenk-Zieger, Julia L. Zimmermann, Gregor Morfill, Sven Becker, Ulrich Harréus, Christian Welz

**Affiliations:** 1 Department of Oral and Maxillofacial Surgery and Facial Plastic Surgery, University Hospital, LMU, Munich, Germany; 2 Department of Otolaryngology, Head and Neck Surgery, University Hospital, LMU, Munich, Germany; 3 Terraplasma GmbH, Garching, Germany; 4 Department of Otolaryngology, Head and Neck Surgery, University Hospital, EKU , Tübingen, Germany; 5 Department of ENT/Head and Neck Surgery, Asklepios Hospital, Bad Tölz, Germany; 6 HNO München Nord, Munich, Germany

**Keywords:** Cold atmospheric plasma, Head and neck cancer, Adjuvant combination therapy

## Abstract

**Background:**

The aim of the present study was to examine the cytostatic effects of cold atmospheric plasma (CAP) on different head and neck squamous carcinoma (HNSCC) cell lines either in isolation or in combination with low dose cisplatin. The effect of CAP treatment was investigated by using three different HNSCC cell lines (chemo-resistant Cal 27, chemo-sensitive FaDu and OSC 19).

**Materials and method:**

Cell lines were exposed to CAP treatment for 30, 60, 90, 120 and 180 s (s). Cisplatin was added concurrently (cc) or 24 h after CAP application (cs). Cell viability, DNA damage and apoptosis was evaluated by dye exclusion, MTT, alkaline microgel electrophoresis assay and Annexin V-Fit-C/PI respectively.

**Results:**

In all cell lines, 120 s of CAP exposure resulted in a significant reduction of cell viability. DNA damage significantly increased after 60 s. Combined treatment of cells with CAP and low dose cisplatin showed additive effects. A possible sensitivity to cisplatin could be restored in Cal 27 cells by CAP application.

**Conclusion:**

CAP shows strong cytostatic effects in HNSCC cell lines that can be increased by concurrent cisplatin treatment, suggesting that CAP may enhance the therapeutic efficacy of low dose cisplatin.

## Introduction

Head and neck squamous cell carcinomas (HNSCC), including cancers of the oral cavity, pharynx and larynx, account for approximately 5% of all cancers worldwide [1]. Primary surgical eradication of the tumor and removal of the regional lymph nodes is the first line therapy in many cases. Subsequently, depending on the pathological staging, a number of patients are eligible for adjuvant treatment with either radiotherapy (RT) or radiochemotherapy (RCT). Especially patients with locally advanced HNSCC, involved resection margins and/or extranodal extension of cancer growth in cervical lymph node metastases should receive concurrent chemoradiation. Studies have shown a local recurrence of 27%-61% as well as regional metastasis as high as 21% and a 5-year survival of 27%—34%, after surgery and adjuvant radiation [2]. However, the use of cisplatin in the standard dose and regime of 20-70 mg/m^2^ every 3 weeks is associated with severe toxicities such as renal- and ototoxicity, and myelosuppression [3, 4]. Numerous HNSCC show intrinsic resistance to platinum drugs [5]. Moreover, many cancers tend to develop multidrug resistance in the course of treatment [6]. Severe side effects of cytostatic treatment frequently lead to incompliance, discontinuation, treatment failure and incompletion of the planned therapy, which increases the risk of cancer recurrence [6]. Novel results hint that low intracellular ROS levels may be the decisive step in cisplatin resistance of cells. In order to decrease side effects, increase therapy efficacy and improve the overall survival rate, recent studies have focused on comparing high-dose cisplatin with cisplatin based combination therapies [7–9].

Cold atmospheric plasma (CAP) is a promising alternative and additional treatment to the current cancer therapies [10, 11]. CAPs are partially ionized gases, which can be generated at atmospheric pressure and operate under room temperature. In this study direct plasma application was used. Both physical and chemical factors in direct CAP treatment have been shown to have an impact on malignant cell viability reduction. Chemical effects have been shown to have the main influence on viability reduction in in-vitro studies. Common cellular responses include the rise of intracellular ROS, DNA damage, as well as mitochondrial and cytoplasmic membrane damage. Several studies have revealed that H_2_0_2_ and NO_2_ are the main reagents associated with CAP exposure of cells. The physical effects include thermal ultraviolet irradiation, and electromagnetic effect and have been shown to be minor in CAP *in-vitro* application in previous studies [12, 13]. Studies have not only proven the anti-microbial efficacy of CAP on human skin and mucosa, but have also proven to be a promising application in the treatment for HNSCC [11, 14–16]. CAP has shown to induce cellular responses such as cell apoptosis, inhibition of growth, selective cancer cell death, DNA damage and/or cell cycle arrest, in this respect being more effective and less toxic than some other common therapies such as radiation and chemotherapeutics in cell line experiments [12, 14]. The toxic effects of CAP on healthy tissues are minor compared to its strong impact on cancer cells in vitro [17, 18].

Unlike many other tumors, HNSCC can be accessed directly via the oral orifice. Thus, a direct CAP application *in-vivo* on the tumor would theoretically be possible. Due to the previously mentioned toxicity of cisplatin, an increase of the systemically applied dose is not possible in extensive or recurrent HNSCC. Therefore, the combination of CAP and cisplatin application could be of clinical relevance. In this study the common cellular responses were detected and analysed by comet assay (DNA damage), MTT assay (mitochondrial damage) and trypan blue staining (cytoplasmic membrane damage). Physical factors of CAP exposure have been shown to be minor in CAP *in-vitro* application in previous studies. The culture temperature of the cell medium in our study was set at 37 °C. Studies have shown that even close CAP exposure will not increase the temperature significantly. Furthermore, the ultraviolet light exposure of cancer cells fails to show significant antiproliferative effects on cancer cells *in-vitro* and therefore physical factors were not specifically addressed in this study [19, 20].

The purpose of the present study was to investigate the effect of CAP treatment (exposure times 30 s, 60 s, 90 s, 120 s and 180 s) on common HNSCC cell lines (FaDu and OSC-19) in comparison to a chemo-resistant HNSCC cell line (Cal 27). A further aim was to examine the therapeutic efficacy of low dose cisplatin in combination with CAP for cisplatin sensitive and resistant HNSCC cell lines *in-vitro*.

### Materials and methods

#### Cell culture

Three different HNSCC cell lines were used for this study. The same two cell lines (OSC 19 (JCBR Cell BANK) and FaDu (ATCC, Manassas, VA, USA)) as in the study of *Welz *et al*.* were used [17, 21] and Cal 27 cell line (ATCC, Manassas, VA, USA), an oral adenosquamous carcinoma cell line which is known to be resistant to *cis*-platinum.

OSC 19 cells were grown in DMEM/Ham´s F-12 (Biochrom AG, Berlin, Germany) and FaDu cells in DMEM (Biochrom). Both cell lines were also supplemented with 10% fetal bovine serum (Gibco, Carlsbad, CA, USA) and 10 U/ml Penicillin–Streptomycin (Biochrom). FaDu cells were additionally supplemented with 1% non-essential amino acids (Invitrogen, Karlsruhe, Germany) and each 1% of L-Glutamine and Sodium Pyruvate (Biochrom). Cal 27 cells were cultured in DMEM also supplemented with 10% fetal bovine serum (Gibco, Carlsbad, CA, USA) and 10 U/ml Penicillin–Streptomycin (Biochrom). All cells were preserved under humidified conditions with 5% CO_2_ at a temperature of 37 °C. All cells were cultured in 25 ml culture flasks (Nunc EasYFlasks) and medium was changed every 2–3 days. For this study, 5 × 10^5^ cells of Cal 27, FaDu or OSC 19 were seeded onto a 6 well plate, and were left to attach for 16 h. For the experiments the cells seeded were splitted at least 3 times.

#### Plasma treatment

**Fig. 1:**
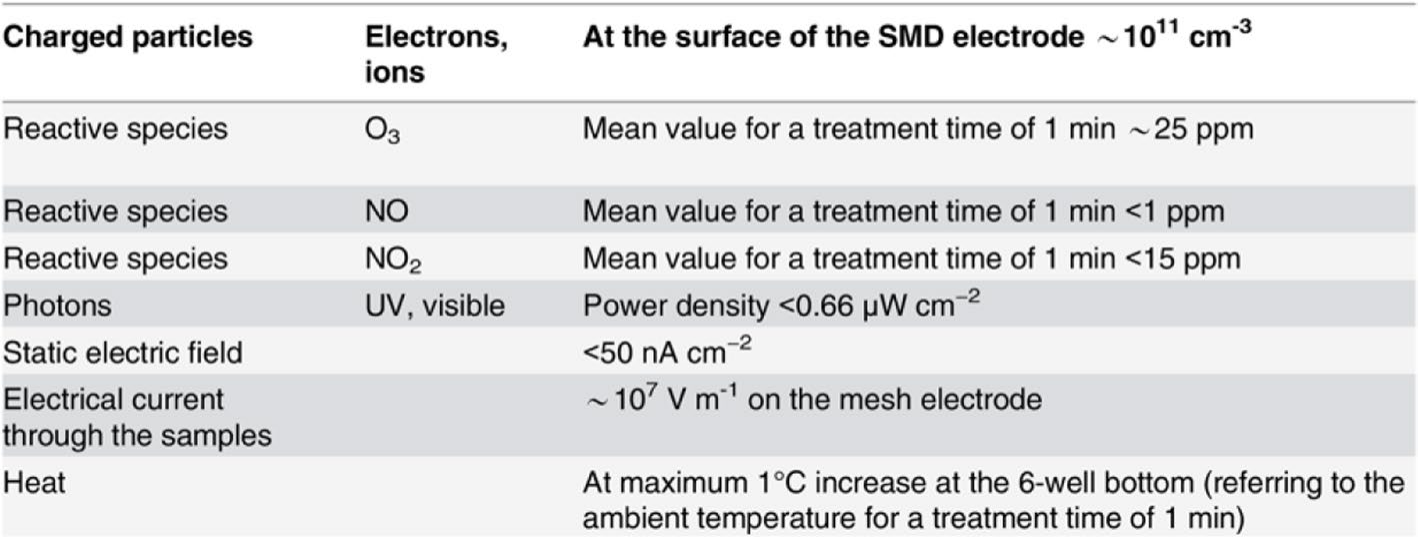
Overview of the characteristics of MiniFlatPlaSter® [17]

**Fig. 2:**
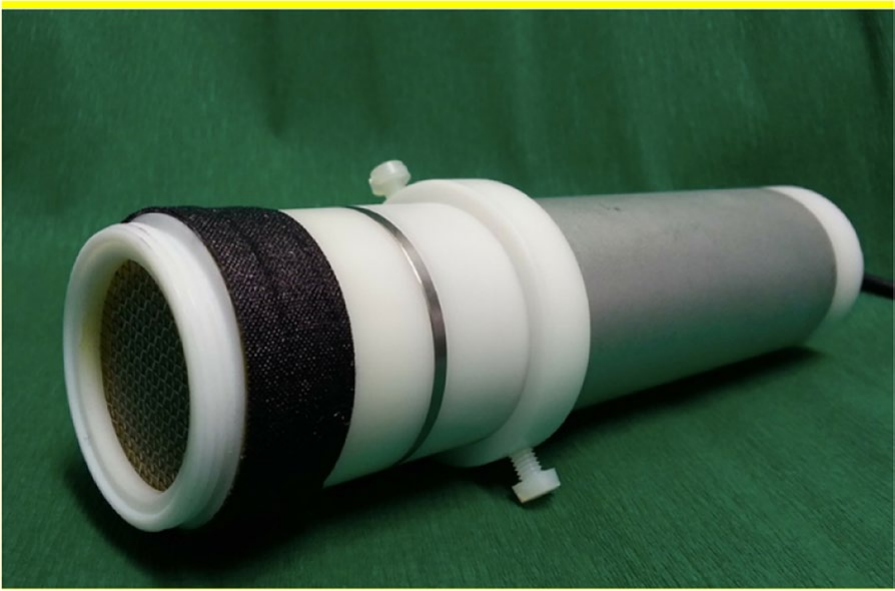
Photograph of the SMD device (MiniFlatPlaSter®) used in this study [17]

**Fig. 3:**
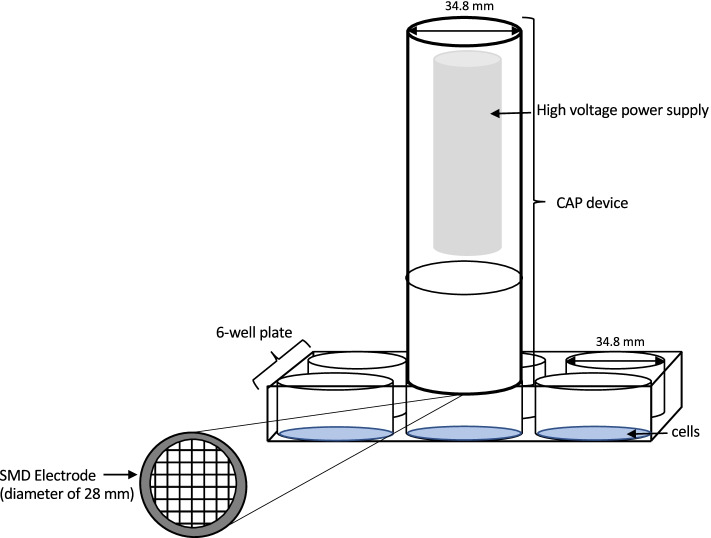
Schematic illustration of the plasma treatment on the different cell types

A CAP device using the surface microdischarge (SMD) technology for plasma production was used for this study [22]. An SMD device is a modified dielectric barrier discharge (DBD) device. The MiniFlatPlaSter® device details, which was used, are described in Maisch et al. [23], Welz et al.[18] and Welz et al. [17] and visualized in Figs. 1, 2 and 3.

A glass epoxy board, which is sandwiched by a stainless-steel mesh grid and copper foil layer creates the SMD electrode. The plasma is produced homogenously on the mesh grid side in the air by applying a high pulse-like voltage of 7 kV with a repetition frequency of 6.75 kHz. Via the mechanism of diffusion, the generated reactive species are transferred to the cells. The MiniFlatPlaSter® produces the plasma indirectly.

One well of a 6-well plate has a rim diameter of 28 mm, fitting the CAP electrode exactly. The medium was then completely removed and the cells were exposed to CAP treatment. The CAP device was placed exactly onto the well rim, causing a closed volume condition (distance between the electrode and cells was 17.5 ± 0.5 mm). The respective controls were treated equally as the plasma treatments except for the CAP exposure. The treatment times used were 30 s, 60 s, 90 s, 120 s and 180 s. Immediately after the appropriate treatment medium was added.

#### Cisplatin treatment

**Fig. 4:**
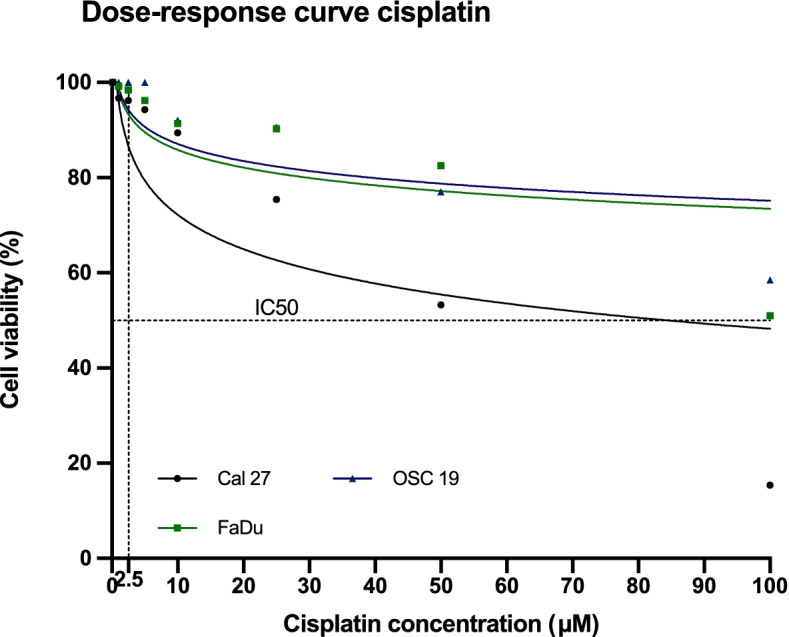
Cisplatin dose–response curve for Cal 27, FaDu and OSC 19 cells, IC_50_ and used concentration of 2.5 µM are displayed

Ten µM cisplatin have been shown to induce sufficient DNA cross-links without inducing apoptosis in cell cultures [21]. The cisplatin concentration of 2.5 µM, which was used in this study, was chosen after analysing the dose–response curve (Fig. 4) of all three different HNSCC cell lines. A low dose of cisplatin was desired with barely any impact on the different HNSCC cells in order to differentiate if a combination therapy could have the same or even greater impact on the cell viability with less cytotoxicity through chemotherapy. Dimethyl sulfoxide (0.05%) (DMSO; Merck, Darmstadt, Germany) was used as a solvent for cisplatin solution preparation. The solution was prepared fresh and protected from light right before the experiments.

Prior to cisplatin application, all cells were seeded and exposed to CAP treatment as stated above in the method and materials section. After the described CAP exposure each cell type was divided into another two treatment groups. For both groups the cells were incubated with 1 ml trypsin/EDTA solution for 8–10 min for trypsination and seeded at 8000 cells/well (100 μl) in a 96 well plate. The first group, cisplatin concurrent (Cis + cc) underwent a 2.5 µM cisplatin application directly after the different CAP treatments. In the second group, cisplatin consecutive (Cis + cs), the cells were incubated (37 °C with 5% CO2), in the 96 well plates in their according medium after CAP treatment and underwent a 2.5 µM cisplatin application after 24 h of incubation (37 °C with 5% CO2). For both groups 2.5 µM of cisplatin was added to each 96 well plate at the stated time point and the well plates were incubated under the same conditions as stated before for another 24 h. Following this, cisplatin was removed and each well plate was washed with PBS. PBS was then discarded and replaced with 10 μl labeling medium containing 0,5 mg/ml MTT and an MTT assay as described in detail below was performed.

#### Cell viability/trypan blue staining (exclusion test)

Trypan blue is used to distinguish between viable and dead cells. The method is based on the principle that the dye is not absorbed by living cells with an intact cell membrane. Dead cells take up the dye due to their damaged membrane and are stained blue [24]. To analyse the cell viability changes after the different CAP treatment times for the different cell types, the trypan blue exclusion test was performed as described by Welz et al. [18]. This was performed for each cell type after CAP treatment (30 s, 60 s, 90 s, 120 s and 180 s). The cells were separated and PBS was discarded after centrifugation (800 rpm for 5 min at 4 ◦ C). The pellets of the treated cells were resuspended in 1 ml of 1 × PBS. 50 μl of cell suspension was then mixed with an equal volume of trypan blue 0.4% (Merck) and transferred to a hemocytometer slide. At least 200 cells were metered for each data point in sixteen microscopic fields. Following that the cells were counted using a light microscope. The percentage of viable cells = ((non-stained cells) / (stained + non-stained cells)) × 100 [17, 18].

#### Cell viability/MTT asssay

﻿The MTT Assay is used to assess cytotoxicity, proliferation and cell viability by measuring the cellular metabolic activity [25]. In this study, the Cell Proliferation Kit I Roche Diagnostics (Roche Diagnostics GmbH, Mannheim, Germany) was used according to the instruction manual as described by Welz et al. [17]. To monitor changes in cell viability after different treatment modalities (CAP, CAP + cc cisplatin application and CAP + cs cisplatin application) and CAP exposure times, the treated cells and the respective controls were trypsinized and seeded at 8000 cells/well (100 μl) in a 96 well plate. The metabolic cell activity was measured 24 h after CAP treatment or CAP + cc cisplatin application or CAP + cs cisplatin application. The culture medium was replaced with 10 μl labeling medium containing 0,5 mg/ml MTT. After 4 h of incubation in a humidified atmosphere (37 °C with 5% CO_2_), 100 μl MTT-staining solution was added to each well followed by an incubation overnight. A VERSAmax™ ELISA- Reader (Molecular Devices GmbH, Biberach, Germany) at a wavelength of 550 nm was used to quantify the purple formazan dye. The reference wavelength corresponds to 690 nm [17, 18]. Every experiment contained triplicate measurements of cell viability reduction of each plasma treatment time and each was repeated five times (measurements for each treatment time *n* = 15). The mean cell viability reduction curves were standardized to the percentage of living cells, whereas the control cells were set at 100%.

#### DNA damage/alkaline microgel electrophoresis (comet assay)

For the detection of DNA damages, the alkaline microgel electrophoresis (Comet assay) was performed after the different CAP treatments. Depending on the extent of damage/fragmentation, the DNA varies in its migration behaviour in an electrical field. Undamaged DNA shows no migration in comparison to fragmented DNA. The higher the fragmentation (damage) the further and faster the migration [26, 27].

The alkaline microgel electrophoresis was carried out as published in the methods and materials section of Welz et. al. [17].

**Fig. 5:**
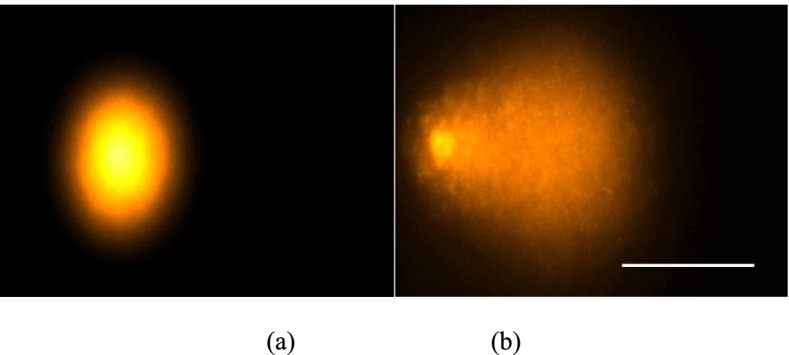
An example of fluorescence images demonstrating the development of tail DNA after CAP treatment (**a**) intact DNA and (**b**) degraded DNA, (horizontal scale bar 100 μm)

After the CAP treatment the cells were incubated with 1 ml trypsin/EDTA solution for 8–10 min for trypsination. Following, neutralization, centrifugation (10 min, 900 U/min), cell counting and cell viability screen with a trypan blue exclusion test were carried out. A two layer agarose was used to ensure stability. The cells were resuspended in 75 µl of 0.7% low-melting agarose (Biozym, Hameln, Germany), applied to slides (Langenbrinck, Emmendingen, Germany) which were covered with normal melting agarose (Biozym) to ensure the stability. The slides were then immersed with alkali solution for 1 h (10% DMSO, 1% Triton-X, 2.5 M NaCl, 10 mM Trizma-Base, 100 mM Na_2_ EDTA and 1% N -lauroylsarcosine sodium salt). After the lysis process, the slides were placed in the gel electrophoresis chamber (Renner, Dannstadt, Germany). Before applying an electric field, they were left with alkaline buffer solution containing 300 mMNaOH and 1 mM Na_2_EDTA at pH 13.2 for 20 min for the DNA double helix to denature. The electrophoresis was started at 0.8 V cm − 1 and 300 mA and continued for 20 min. The slides were then neutralised with Trisma base, 400 mM, pH 7.5 (Merck, Germany). After this, they were stained with 75 μl ethidium bromide (Sigma; [51 μM]) and analysed with a DMLB microscope (Leica, Bensheim, Germany). By random pattern, 80 cell nuclei per slide (2 slides per CAP treatment time) were selected and digitized with the attached monochrome CCD camera (Cohu Inc., San Diego, CA, USA). Using the image analysis software Komet +  + (Kinetic Imaging, Liverpool, UK) DNA-migration was measured. Cells containing damaged DNA have the appearance of a comet under the microscope, where the undamaged DNA is the „head “ and the damaged DNA is the „tail “ of the comet (Fig. 1 (a-b)). Intact DNA is shown as an intact nucleus, „head “, with no tail. For orientation of genotoxicity the Olive Tail Moment (OTM) was used and automatically calculated by the computer software (OTM = (tail mean-head mean) x % of DNA in the tail) [28]. Cells which showed an OTM < 2 were considered as undamaged [29]. The migration was measured by the software and calculated by the % of DNA tail. This is the relative fluorescence intensity in the “head” and “comet tail” [30] (Fig. 5).

#### Apoptosis/Annexin V Fit-C

**Fig. 6:**
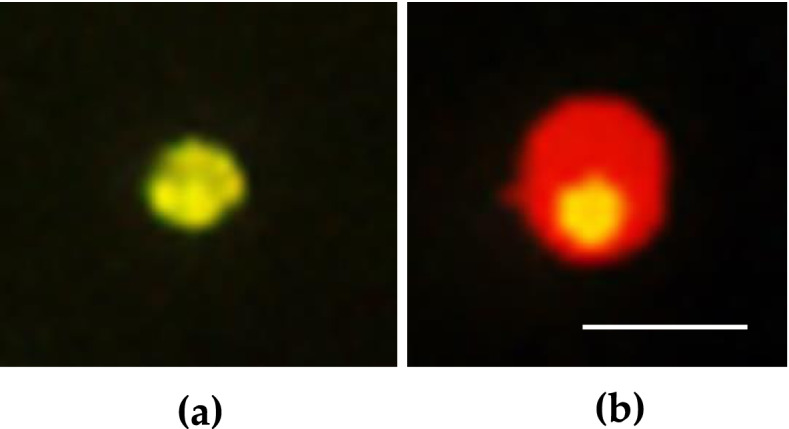
(a-b) An exemplary fluorescence microscopy image of Annexin V/PI stained cells (**a**) green fluorescence showing early apoptotic cells, (**b**) red fluorescence showing late apoptotic/necrotic cells) (horizontal scale bar 50 µm)

Annexin V Fit-C is a rapid and sensitive method to determine apoptotic from necrotic cells. 24 h after CAP treatment the induction of apoptosis was investigated with fluorescence microscopy using an Annexin V Fit-C detection kit (PromoKine, Heidelberg). CAP treatment and trypsinisation were carried out as described above. HNSCC cell lines were stained with Annexin V Fit-C and propidium iodide (PI) according to the manufacturer’s instructions. In this case, 1 × 10^5^ cells were resuspended in 500 μl binding buffer solution, incubated for 5 min under red light with 5 μl Annexin V-FITC and 5 μl propidium iodid (PI). Phosphatidylserin (PS) residues are found on the inner surface of the membrane in normal cells, hence being inaccessible to Annexin V. An early step of apoptosis is the translocation of phosphatidylserin (PS) from the internal to the external face of the plasma membrane as the cell membrane becomes permeable. Annexin-V is a Ca 2^+^ phospholipid binding protein with a high affinity to PS and can therefore be used as a sensitive marker for the apoptotic cells. Combining Annexin-V labelling with PI staining allows discriminating between (early) apoptotic and late apoptotic/necrotic cells, as PI will be able to stain the DNA in the nucleus. One hundred cells per CAP treatment were counted with the help of a hemocytometer and early apoptotic cells were identified with fluorescence microscopy, and indexes calculated out of 100 cells (Fig. 6).

#### Statistical analysis

Unless stated otherwise, the data is presented as the arithmetic mean ± 95% CI. Graph Pad Prism 7.0. Software was used for statistical analysis. The Two-way ANOVA test with a Bonferroni correction test as a post-test, to counteract the problem of multiple comparisons, was used to calculate the statistical significance of the results. Differences were considered as significant at the calculated stated adjusted *p*-values, prior the statistical analysis.

## Results

### Trypan blue staining

Changes in cell viability reduction in the three different cancer cell types were measured by trypan blue staining after CAP treatment. FaDu and OSC 19 already showed a significant viability reduction after 90 s of CAP treatment, 86% and 70.6% respectively. All cells showed a significant decrease in cell proliferation after CAP exposure for 120 s (average viable cells after 120 s: Cal 27 86.6%, FaDu 63.9%, and OSC 19 47.0%).

### MTT viability assay

**Fig. 7:**
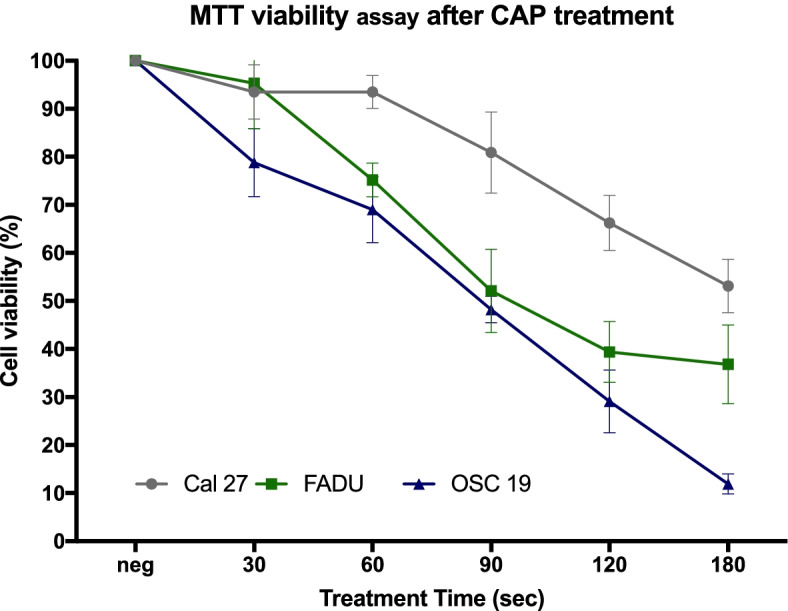
Detection of cell viability (%) with an MTT assay for all three cell types at different CAP treatment times. Line graph is used to present the results (mean and error bars 95% CI is shown for all three cell lines, (for each treatment time per cell type *n* = 15)

Figure 7 shows the reduction in the viability of Cal 27, FaDu and OSC 19 cell lines after CAP treatment for 30 s, 60 s, 90 s, 120 s and 180 s. The mean cell viability reduction curves were standardized to the percentage of living cells, control cells were set at 100% to allow the visual comparison between all cell lines. For the statistical analysis the absorbance values were compared.

Figure 7 displays that all cell lines showed a significant decrease of viable cells, in a dose dependent manner. The FaDu and OSC 19 cell line showed significantly lower absorbance values and therefore a significant decrease of viable cells compared to the Cal 27 cell line for all exposure times (*p* < 0.001). Both FaDu and OSC 19 had a similar cell survival after 60 s and 90 s (75.2% vs 69.0% and 52.1% vs. 48.2% respectively). OSC 19 showed the strongest reduction of cell viability reduction at 120 s and 180 s of CAP treatment compared to the other two cell lines (*p* ≤ 0.0207). Cal 27 cells showed the weakest reaction to CAP. After 180 s the mean number of viable cells was 53.1%. The half maximal inhibitory dose (IC_50_) was reached after 90 s of exposure in OSC 19 and after 120 s in FaDu cells.

### DNA damage after CAP treatment

In order to evaluate the DNA damage in the three different cell types after CAP treatment the alkaline gel electrophoresis was performed. Table 1 shows the average OTM values of the different cancer cell types after the different times of CAP treatment. An OTM value > 2 was considered to show DNA damage.

**Table 1:** Average OTM values of the different cell types at the different treatment times with CAP

Cell Line	30 s	60 s	90 s	120 s	180 s
Cal 27	1.32	3.07	2.78	2.95	4.78
FaDu	1.64	3.03	6.87	9.28	10.60
OSC 19	1.48	3.05	4.82	6.11	7.69

**Fig. 8:**
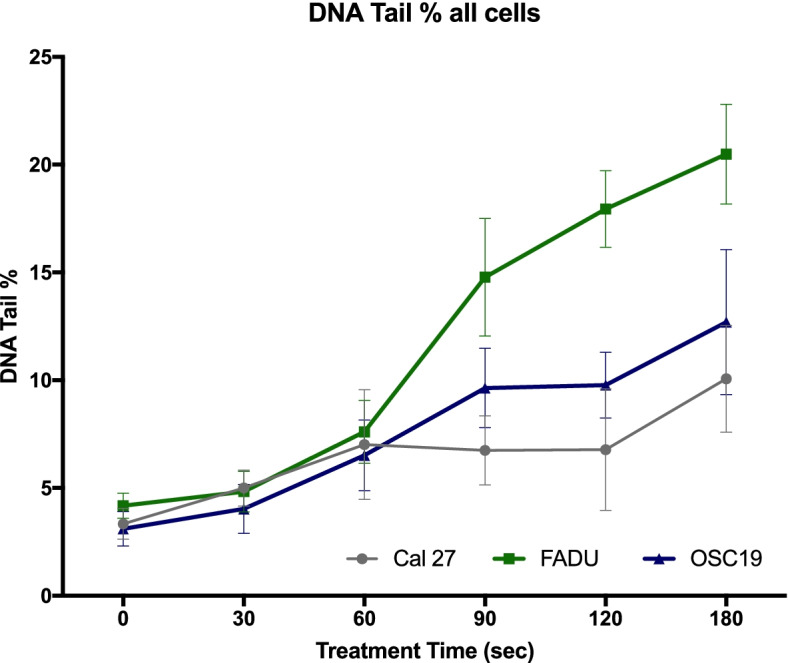
Detection of DNA Damage (comet assay) for all three cell types. Line graph is used to present the results (mean and error bars 95% CI is shown for all three cell lines, (for each treatment time per cell type *n* = 15)

Figure 8 shows the DNA damage of all three different cell lines after CAP treatment for 30 s, 60 s, 90 s, 120 s and 180 s. The DNA damage was quantified by the DNA tail (%). It is the relative fluorescence intensity in the “head” (undamaged DNA) and “comet tail” (damaged DNA). These values were used for statistical analysis. All cell lines showed the first significant increase in DNA damage after 60 s of CAP treatment compared to the control group (*p* ≤ 0.0366), (Tables 2, 3, and 4). Only the FaDu cells showed a continuous significant cell damage increase by further exposure to CAP (significant difference between 30 s vs 60 s, 60 s vs 90 s and 90 s vs 120 s) (Table 3). Solely the Cal 27 cells showed no significant increase in cell damage between the treatment times 60 s (97.6%), 90 s (97.2%) and 120 s (86.6%) (Table 2). However, they showed late DNA damage increase between CAP treatment times of 120 s and 180 s *(p* ≤ 0.0171).

**Table 2:** Adjusted *p*-values (Bonferroni’s multiple comparisons test) for cell damage (DNA tail %) with the comet assay of Cal 27, level of significance *p* ≤ 0.0366

	**30 s**	**60 s**	**90 s**	**120 s**	**180 s**
*Sig vs. Control*	> 0.9999	0.00046	0.012	0.015	< 0.0001
*Sig vs 30 s*		0.6152	> 0.9999	> 0.9999	< 0.0001
*Sig vs 60 s*			> 0.9999	> 0.9999	0.0366
*Sig vs 90 s*				0.2048	< 0.0001
*Sig vs 120 s*					< 0.0001

**Table 3:** Adjusted *p*-values (Bonferroni’s multiple comparisons test) for cell damage (DNA tail %) with the comet assay of FADU, level of significance *p* ≤ 0.0259

	**30 s**	**60 s**	**90 s**	**120 s**	**180 s**
*Sig vs. Control*	> 0.9999	0.0108	< 0.0001	< 0.0001	< 0.0001
*Sig vs 30 s*		0.0823	< 0.0001	< 0.0001	< 0.0001
*Sig vs 60 s*			< 0.0001	< 0.0001	< 0.0001
*Sig vs 90 s*				0.0259	< 0.0001
*Sig vs 120 s*					0.1623

**Table 4:** Adjusted *p*-values (Bonferroni’s multiple comparisons test) for cell damage (DNA tail %) with the comet assay of OSC 19, level of significance *p* ≤ 0.0355

	**30 s**	**60 s**	**90 s**	**120 s**	**180 s**
*Sig vs. Control*	> 0.9999	0.012	< 0.0001	< 0.0001	< 0.0001
*Sig vs 30 s*		0.1912	< 0.0001	< 0.0001	< 0.0001
*Sig vs 60 s*			0.0294	0.0189	< 0.0001
*Sig vs 90 s*				> 0.9999	0.0355
*Sig vs 120 s*					0.0543

The first significant difference of DNA damage between all three cell lines was seen after 90 s of CAP treatment time (Fig. 8). The FaDu cell line showed the highest DNA damage (14.78%) compared to Cal 27 (6.74%) showing the least and OSC 19 cells (9.63%) at this treatment time. There was a further difference in DNA damage to be seen at treatment time points 120 s and 180 s between all cell types (Fig. 8). The FaDu cell lines were measured to continuously have the significantly highest cell damage (17.94% and 20.48% respectively) compared to Cal 27 and OSC 19 at these time points.

### Apoptosis

**Fig. 9:**
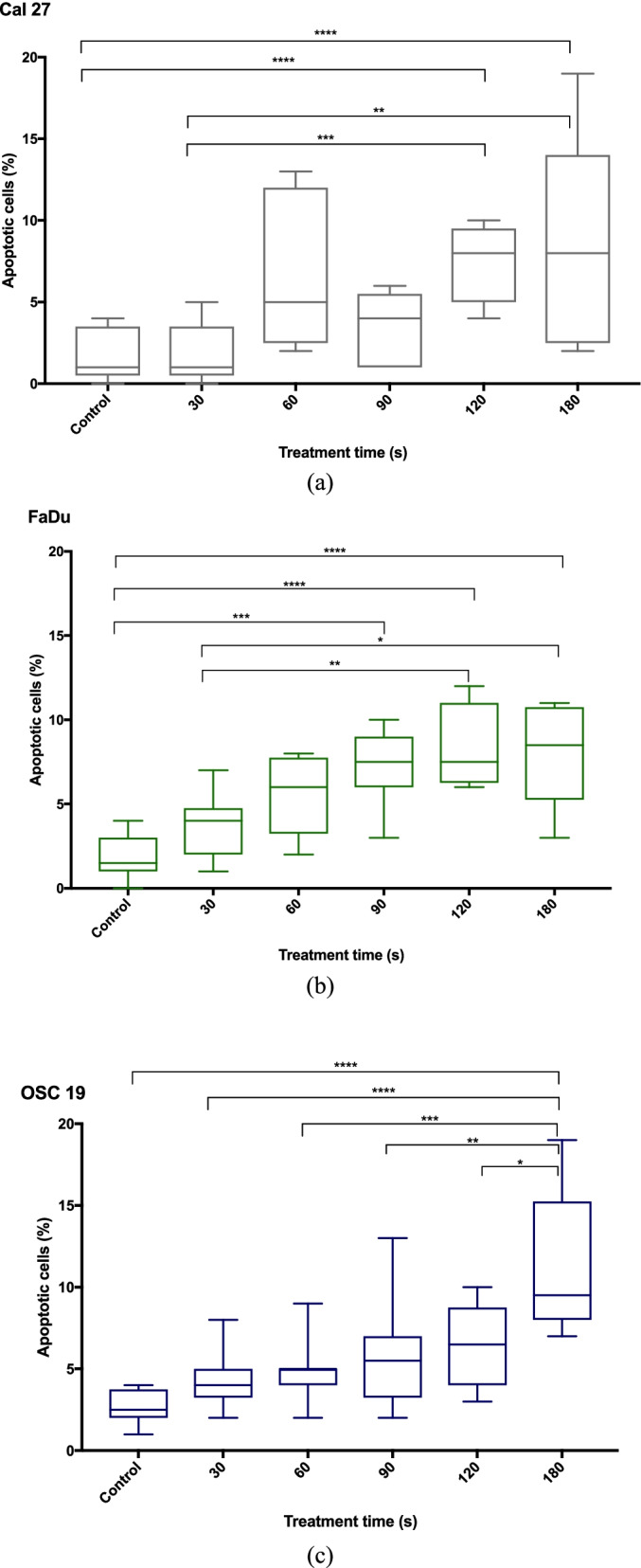
Detection of apoptotic cells for each cell line (Fig. 9a: Cal 27, Fig. 9b: FaDu, Fig. 9c: OSC 19) after the different CAP exposure times (for each treatment time per cell type *n* = 15)

Annexin V Fit-C/PI staining was performed 24 h after CAP treatment in order to clarify if the time dependent increase of DNA damage induces apoptosis and if reduction in cell viability can be explained by programmed cell death. Figure 9(a-c) shows the number of apoptotic cells for each cell line after the different CAP exposure times.

Cal 27 and FaDu showed a significant increase in apoptosis after 120 s and 90 s CAP exposure respectively (*p* < 0.001). OSC 19 showed a significant percentage of apoptotic cells after 180 s of CAP treatment (*p* < 0.0001). OSC 19 showed the highest mean percentage of apoptosis (11.25% at 180 s) in comparison to the FaDu (8.50% at 120 s) and Cal 27 (8.20% at 180 s).

### Cell viability after cisplatin application

**Fig. 10:**
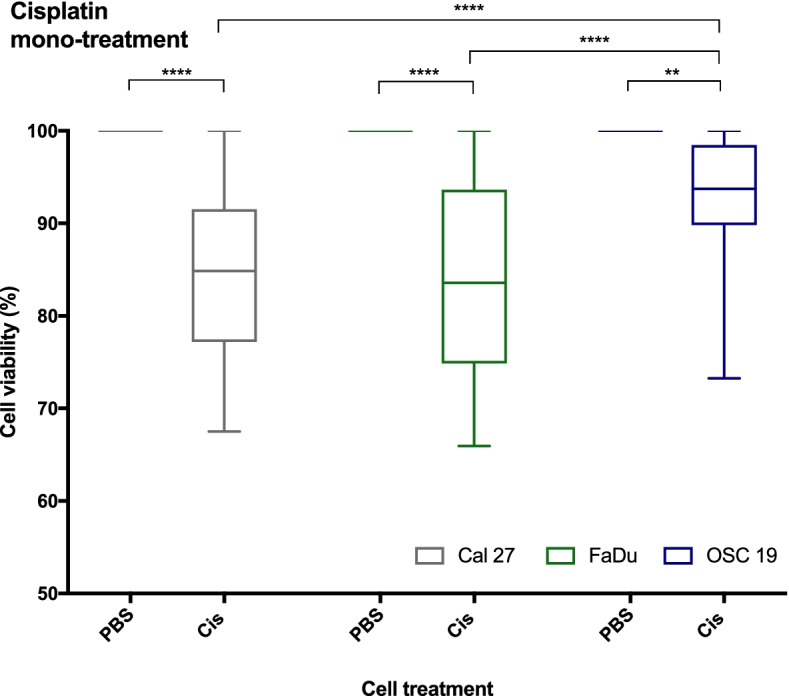
Detection of cell viability (%) with an MTT assay for all three cell types after cisplatin treatment. Mean and error bars 95% CI is shown for all three cell lines, (for each treatment time per cell type *n* = 15)

Figure 10 shows the cell viability of the three cell types after Cisplatin mono treatment. All cell lines showed a significant reduction in cell viability (*p* ≤ 0.0063). The mean cell viability was 83,87%, 83.0% and 93.03% for Cal 27, FaDu and OSC 19 respectively. The OSC 19 cell line showed a signifcantly higher cell viability after cisplatin monoapplication compared to the other two cells (*p* < 0.0001).

### Cell viability after CAP treatment and cisplatin application

All cell types showed a significant further reduction of cell viability with a combination treatment compared to mono treatment with cisplatin. Cal 27 and FaDu already showed a significant cell viability reduction compared to a mono treatment after 30 s CAP + cis cc (*p* < 0,0001). The Cal 27 cell line showed a significant difference between mono and consecutive cisplatin treatment a prior 180 s CAP exposure (*p* < 0,0001). FaDu cells already showed a significantly lower cell survival at 90 s CAP + Cis cc compared to a cisplatin monotreatment. The OSC 19 cell line presented similar results in cell viability in a combination therapy compared to a mono cisplatin treatment. CAP + Cis cc showed a significant cell viability reduction after 90 s compared to mono chemotherapy and CAP + Cis cs already after 60 s (*p* < 0.0001).

**Fig. 11:**
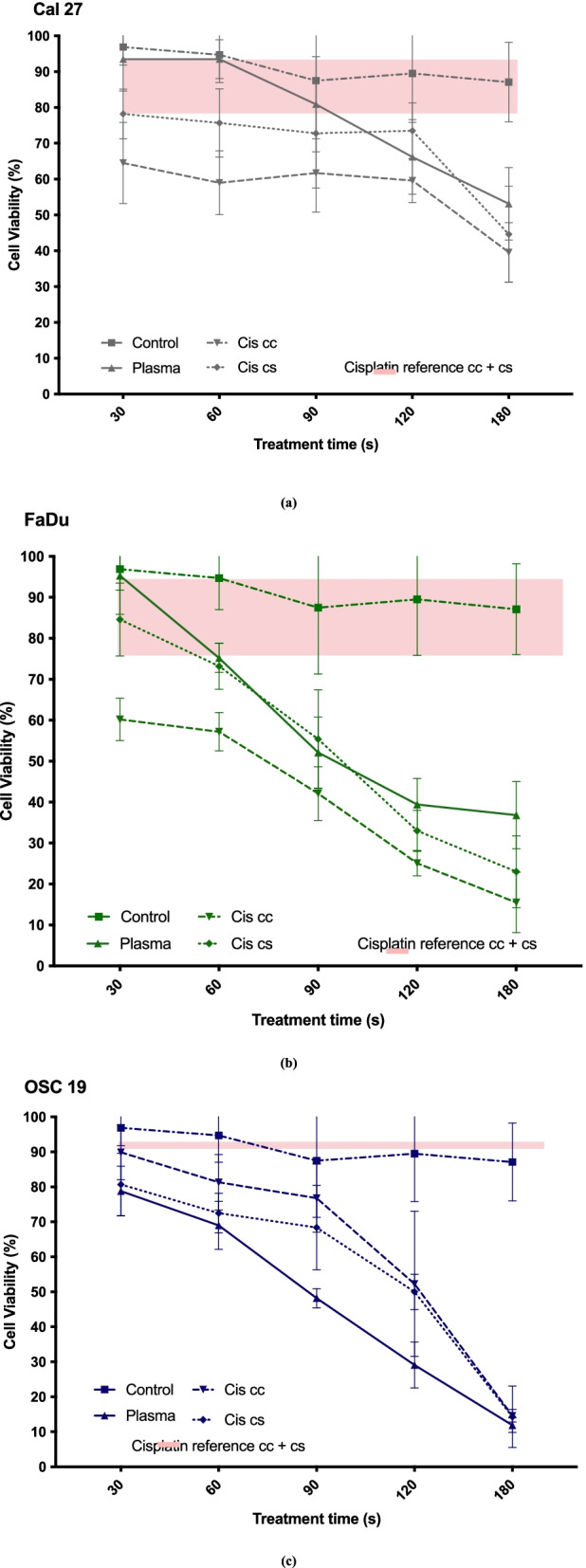
Detection of cell viability (%) with an MTT assay for the different treatment types and times for each of the individual cell types and the cisplatin reference value, (Fig. 11a: Cal 27, Fig. 11b: FaDu, Fig. 11c: OSC 19). Line graph is used to present the results (mean and error bars 95% CI is shown for all three cell lines, (for each treatment time per cell type *n* = 15)

Figures 11(a-c) show the cell viability reduction of Cal 27, FaDu and OSC 19 cell lines after solely Cisplatin treatment, CAP treatment, CAP treatment + Cisplatin concurrent (Cis cc) application and CAP treatment + consecutive (Cis cs) application for 30 s, 60 s, 90 s, 120 s and 180 s and the control group. The cisplatin reference value is displayed on the graphs for each cell line. The mean cell viability reduction curves were standardized to the percentage of living cells, the controls were set at 100% to allow the visual comparison between all cell lines. For the statistical analysis the absorbance values were used and analysed.

The cell line Cal 27 showed significant difference in cell viability at almost all treatment time points. At treatment times of 30 s and 60 s there was a significant difference for all examined treatments (CAP vs CAP + Cis cc, CAP vs. CAP + Cis cs and CAP + Cis cc vs. CAP + Cis cs). At 90 s no difference in cell survival could be seen between CAP and CAP + Cis cs. However, CAP + Cis cc showed a significantly lower cell viability at 90 s compared to the other treatments *(p* ≤ 0.0237). When comparing the different treatment groups, the significantly lowest cell survival was measured at all time points for the treatment with CAP + Cis cc application for the Cal 27 group except at 180 s *(p* ≤ 0.0237). An additional cisplatin treatment (concurrent and consecutive) after CAP treatment showed an additive effect on cell viability reduction to solely cisplatin or CAP therapy after only 30 s of CAP exposure time. The Cal 27 cell line only showed a significant difference between a mere CAP treatment and cisplatin treatment after 180 s CAP exposure. In both treatment groups the cell viability was > 80% at all other treatment times.

The FaDu cells showed a difference in cell survival at all treatment times between only CAP treatment and CAP + Cis cc treatment except after 90 s. A significantly higher cell reduction was to be seen by the additional Cis cc application for treatment times at 30 s and 60 s (*p* ≤ 0.0015). There was no significant difference in viability to be seen for solely CAP treatment vs CAP + Cis cs application except at 180 s (*p* > 0.05).

The OSC 19 cells had a significant difference in the amount of cell survival between solely a CAP treatment, CAP + Cis cc application and CAP treatment + Cis cs application at 90 s and 120 s (*p* ≤ 0.0027). Here an additive effect was seen with the addition of low dose cisplatin cc and cs. There was no difference at these time point in cell viability between CAP + Cis cc and CAP + Cis cs. However, all three treatments showed lower cell viability at all treatment times compared to the cisplatin reference value.

The adjusted *p*-values are presented in Table 5 with a level of significance at *p* ≤ 0.0221 for CAP + Cis cc and *p* ≤ 0.041 for CAP + Cis cs. The cell viability was significantly lower after CAP + Cis cc application within the Cal 27 cell line compared to the OSC 19 group after treatment times 30 s (64.5% and 89.9% respectively), 60 s (59.0% and 81.3% respectively), and 90 s (61.7% and 76.8% respectively). The FaDu cells also showed a significantly higher cell reduction with an additional cisplatin cc treatment compared to OSC 19 at treatment times 30 s (60.2%), 60 s (57.2%), 90 s (42.1%) and 120 s (25.1%). FaDu cells measured a significantly lower cell viability compared to the Cal 27 cell line after CAP exposure times 90 s, 120 s and 180 s and Cis cc treatment.

**Table 5:** Adjusted *p*-values (Bonferroni’s multiple comparisons test) for cisplatin applications comparison of different cell types, level of significance for CAP + Cis cc, *p* ≤ 0.0221 and for CAP + Cis cs, *p* ≤ 0.041

Adjusted *p*- value for CAP + Cis cc application						Adjusted *p*- value for CAP + Cis cs application				
Cell lines	**30 s**	**60 s**	**90 s**	**120 s**	**180 s**	**30 s**	**60 s**	**90 s**	**120 s**	**180 s**
Cal 27 vs. FaDu	0.0119	> 0.9999	< 0.0001	< 0.0001	< 0.0001	0.5175	> 0.9999	0.0032	< 0.0001	0.0003
Cal 27 vs. OSC 19	0.0012	0.0001	0.0011	0.801	< 0.0001	> 0.9999	> 0.9999	> 0.9999	< 0.0001	< 0.0001
FaDu vs. OSC 19	< 0,.001	< 0.0001	< 0.0001	< 0.0001	> 0.9999	> 0.9999	> 0.9999	0.0413	0.0041	0.453

The treatment with CAP treatment + Cis cs application showed a significantly lower cell survival after 90 s in the FaDu cell line (55.4%) compared to the Cal 27 (72.8%) and OSC 19 (68.4%) group. The duration of 120 s CAP exposure + cisplatin cs application presented significantly less viable cells in the FaDu cell group compared to Cal 27 (73.5%) and OSC 19 (50%), whereas Cal 27 had significantly higher survival compared to both other cell lines. After the CAP exposure time of 180 s + Cis cs application all cell lines show an average of less than 50% cell viability. The FaDu and OSC 19 cells lines measure a significantly higher cell reduction at this treatment time than the Cal 27 (23.0% and 14.3%, respectively).

## Discussion

In literature, CAP is one of the most promising new approaches to cancer therapy. Several studies have shown a successful application as an alternative therapy for malignant tumors [12, 31, 32]. CAP may be applied as a primary treatment tool or could be used to improve the response of malignant tumor cells to chemotherapy or radiation. The overall 5-year survival rate in HNSCC is approximately 50% [2]. To improve outcomes, more effective single and combined therapies are needed because the conventional therapies used today still show too many limiting factors. A combination therapy with cisplatin and CAP exposure could be a promising alternative concomitant treatment for advanced HNSCC, in particular for those showing a cisplatin resistance. Furthermore, a reduction of toxicity could be achieved by dose reduction of cisplatin application. Systemic cytotoxicity and intrinsic/acquired chemo-resistance are limiting factors. It leads to premature termination of the planned therapy, treatment failure and even cancer recurrence is often the case.

The aim of the present study was to analyze the effects of CAP on cell viability and toxicity of HNSCC cells in vitro either in isolation or in combination with low-dose cisplatin. Further aims were to test these effects on cells with respect to different exposure intervals to CAP and application times of low dose cisplatin.

This study found that CAP has a cytotoxic effect on all three different HNSCC cells with increasing impact at longer exposure times. It is interesting to note that the inhibitory effects of CAP (alone or in combination with cisplatin) varied with respect to the cell line. Faster proliferating cells (OSC 19) showed a better response to CAP mono treatment than the other two cell lines, FaDu and Cal 27 respectively. This effect can be enhanced by combining CAP application with low – dose cisplatinum treatment, especially on slower doubling times (Cal 27 and FaDu). A combined treatment CAP + cis cc showed a significantly lower cell viability in the chemo-resistant Cal 27 compared to any of the mono treatments for this cell line.

The results are of clinical importance because the combination of topical CAP treatment in combination with systemic cisplatin administration could reduce the standard chemotherapy drug dosage currently administered and hereby reduce toxicity, side effects and improve prognosis. Additionally, a combination therapy could also improve the response of chemo-resistant HNSCC.

Generally, as discussed by Keidar et al., the cellular response to CAP exposure is dependent on the individual cell line [31]. Different HNSCC cell lines react differently to CAP treatment, the cytotoxic effect of CAP varies. The cytotoxic effect is a function of DNA damage induced in the targeted cells. Since DNA damage is not directly observable, the present study used the Comet assay to detect DNA damage. Trypan Blue and MTT assay were performed to quantify the reduction in cell viability. Here, all three HNSCC cell lines showed a CAP-dose dependent increase of DNA damage and a cell-viability reduction. The half-maximal inhibitory concentration (IC50) was reached after 90 s for OSC-19 cells and after 120 s for FaDu cells, respectively. In Cal 27 cells, a significant decrease of cell viability could be reached after 60 s. However, even with an exposure of up to 180 s, a reduction of cell viability below 50% was not possible. These results are in agreement with Welz et al., who demonstrated dose dependent cytotoxic effects in OSC 19 and FaDu cell lines and a general effectiveness of CAP against HNSCC cells [17, 33].

We carried out AnnexinFit-C/PI staining in order to evaluate if dose dependent DNA damage induces apoptosis. All three HNSCC cell types showed a significant increased percentage of apoptotic cells after 180 s of CAP treatment compared to their control. Again, the higher proliferating cells (OSC 19) showed a higher amount of apoptotic cells after 180 s than the slower proliferation cells (FaDu and Cal 27). The different vulnerability of the cell lines towards CAP treatment may be explained by their cell cycle and doubling time. The most significant effect of CAP was observed in the fast-doubling OSC-19 (approx. < 25 h) and FaDu (approx. 30 h) cell lines, while Cal 27 (approx. 45 h) were affected the least [34–38]. The cell growth rate has been proven to play a significant role in the cells sensitivity towards radiotherapy. A higher growth rate/faster doubling time has been shown to cause more cell reduction/cell growth arrest than slower growing cell lines. This was also observed in our study for cell vulnerability towards CAP exposure.

Volotskova et al. showed in their study that cells are more likely to be damaged by CAP treatment during S-phase of the cell cycle [39]. This is supported by our findings, as FaDu and OSC-19 cell lines with a faster doubling time, hence having a higher number of cells in the S-phase of the cell cycle, showed a higher susceptibility towards CAP. As already shown by Welz et al. [17] it can be assumed that there are similarities between the anticancer mechanisms of radiation as high DNA damage and cell death can be observed in high proliferating cells after CAP treatment, whereas low proliferating cells seem to be less affected by the same CAP application.

In addition to the primary cytotoxic effect of CAP, we evaluated the response of malignant epithelial tumor cells with an additional cisplatin application. All epithelial cell lines showed a significant cell viability reduction after a mono treatment with cisplatin. Even Cal 27 cells which are supposedly resistant to chemotherapy with cisplatin [36, 37]. In the present study, the vulnerability of Cal 27 towards treatment with cisplatin was increased by the prior application of CAP. This was highly significant for all exposure times. This suggests that a prior CAP treatment could increase chemo-sensitivity. This assumption is supported by Köritzer et al., who demonstrated an increase of temozolomide efficiency in resistant Glioblastoma cells following CAP treatment [40]. Here, a 30 s exposure was sufficient to induce chemo-sensitivity in an otherwise resistant cell line. Further, other publications have also shown a successful CAP treatment on cancer cells resistant to current treatments as well as possible dose reductions [41].

Cal 27 also showed a very low cell viability reduction after CAP treatment for all treatment times. Even after 180 s of CAP exposure mean cell viability was still 53.1%, showing a possible CAP-resistance. CAP exposure can lead to a reduction in redox regulators. This could be an explanation for chemo-sensitivity induction in the Cal 27 cell line. Furthermore, the theory of cell activation/sensitization, a unique feature of direct plasma application in comparison to indirect CAP treatment, could also be an explanation. Prior studies have shown that cancer cells show a different resistance towards reactive species, resulting in these cells remaining in an activation or sensitization state without viability reduction for approx. 5 h [13]. Cal 27 showed the greatest CAP resistance in our results. However, our results suggest that they could be sensitized to cytotoxicity with CAP exposure towards low-dose cisplatin as a significantly higher cell viability reduction was achieved by the concurrent chemotherapy application.

Novel results imply that low intracellular ROS levels may be the decisive step in cisplatin resistance of cells [42]. This study was designed to examine the interactions between combined CAP and cisplatin exposure in chemo-resistant and -sensitive HNSCC cells. The theory was, that direct CAP treatment of HNSCC cells increases the intracellular ROS levels so that cisplatin sensitivity can possibly be increased or restored. Cisplatin resistance of cells is a very complex and multifaceted characteristic [43]. One important step might be the detoxification of cisplatin by ROS scavengers like glutathione (GSH). It is highly likely that the main antioxidant effect of N-acetylcysteine (NAC) is due its cysteine element which might supplement intracellular GSH levels. On the other hand, there is controversy whether NAC can directly increase intracellular GSH levels. Some authors agree that the intracellular desulfuration of NAC provides the antioxidant effect. However, further studies are needed for the pdetermination of the decisive ROS scavenger for CAP/cisplatin.

Only Cal 27 cells showed a difference in cell reduction between a concurrent and consecutive cisplatin application after CAP exposure. An explanation could be that there was enough time for DNA reparation after minimal CAP damage and therefore no additive effect when applying cisplatin 24 h after CAP exposure. Only direct application showed an additive effect. The other cells (FaDu and OSC-19) showed no significant difference between cisplatin cc or cs applications, most likely because of maximum DNA damage already caused by only CAP exposure. These results could again be related and explained by the different doubling times of the cell lines. The faster doubling cell lines showed no difference in the cell viability results between the application times of cisplatin after CAP treatment, whereas the slower doubling Cal 27 cells showed a different sensitivity towards the time of cisplatin application after CAP therapy.

In this study, only 30 s of CAP exposure combined with a single cisplatin cc application showed a mean cell viability of 64.5% in the otherwise chemo-resistant Cal 27 cell line. Studies have implied that agents with antioxidant activities could have a chemoprotective effect on healthy tissue against chemotherapy [44, 45] and implied the potential selectivity of CAP towards malignant cells [11, 15, 46]. Welz et al. showed no cytotoxic or mutagenic effect on healthy mucosal tissue for CAP treatment times below 120 s, suggesting CAP being a selective agent for malignant cells [18]. Guerrero-Preston et al. also showed a significant cell viability reduction using using CAP of a HNSCC cell line in vitro with only minimal impact on normal oral cavity epithelial cell lines [15]. This could be a promising prospect for a combined chemo- and CAP treatment [47].

Additionally, the chemo-sensitive cells FaDu and OSC 19 also showed lower cell viability when exposed to CAP and concurrently treated with cisplatin. In both cases 60 s of CAP treatment + Cis cc application showed a noticeable increased cell viability reduction when comparing to the cisplatin reference and only CAP exposure (FaDu > OSC 19). Again, a CAP treatment time reduction in combination with a single cisplatin application should be taken into consideration. This way, one could achieve a cisplatin dose reduction even in known chemo-sensitive tumor cells.

The following limitation of the study have to be discussed. The chemoresistance to cisplatin of Cal 27 cells used was not significant which may be a result of an altered microenvironment. Exposure of these cells to increasing concentrations of cisplatin prior to CAP treatment and more precise control of the microenvironment (e.g. pH) to generate more reliable drug-resistant cells should be considered in further studies to re-evaluate the chemo sensitivity induction through CAP. However, other preclinical studies have shown that Cal 27 cells are not totally resistant to cisplatin and that the resistance is dose-dependent [48]. A SMD plasma device (MiniFlatPlaSter®), a direct plasma source was used. Indirect plasma devices, an example being a plasma jet are predominantly used for CAP studies. Furthermore there is also a difference to be made in the application method of CAP, direct and indirect treatment [46]. Both types of devices (direct and indirect) have shown their success in selectively reducing cell viability and inducing DNA damage in head and neck malignant tumor cells. Nonetheless, the sensitivity of these cells to the devices is yet to be established. In addition, further studies have to examine and evaluate the potential superiority of the different systems and treatment types over the other in the application for HNSCC. This needs to be taken into consideration when comparing CAP results with each other.

## Conclusions

In conclusion, this study demonstrates a decrease in cell viability in all three HNSCC cells types solely to CAP exposure, especially for treatment times 90 s and more. However, the supposedly cisplatin resistant cell line Cal 27 showed the least response to a CAP mono therapy. All HNSCC cell lines showed a CAP-dose dependent DNA damage as well as a significant increase in apoptotic cells with longer CAP exposure times. We observed an additive effect within all cell types, in particular for short CAP treatment times and an immediate subsequent cisplatin application. Especially Cal 27 cell line showed an increased vulnerability to treatment with cisplatin by the prior application of CAP for all exposure times, suggesting a possible chemo-sensitivity induction by a primary CAP treatment of only 30 s. Finally, we demonstrated the successful application of a direct plasma source for the treatment for HNSCC chemo-sensitive and –resistant carcinoma cells. Additionally, the study presents the potential of CAP and cisplatin combination therapy as a possible addition to adjuvant therapy options with low dose chemotherapy for the treatment of HNSCC.

## Data Availability

All data generated or analysed during this study are included in this published article.
