# Correction: Primary cold atmospheric plasma combined with low dose cisplatin as a possible adjuvant combination therapy for HNSCC cells—an in‑vitro study

**DOI:** 10.1186/s13005-023-00386-x

**Published:** 2023-08-18

**Authors:** Teresa F. Brunner, Florian A. Probst, Matthias Troeltzsch, Sabina Schwenk‑Zieger, Julia L. Zimmermann, Gregor Morfill, Sven Becker, Ulrich Harréus, Christian Welz

**Affiliations:** 1 Department of Oral and Maxillofacial Surgery and Facial Plastic Surgery, University Hospital, LMU, Munich, Germany; 2 Department of Otolaryngology, Head and Neck Surgery, University Hospital, LMU, Munich, Germany; 3 Terraplasma GmbH, Garching, Germany; 4 Department of Otolaryngology, Head and Neck Surgery, University Hospital, EKU, Tubingen, Germany; 5 Department of ENT/Head and Neck Surgery, Asklepios Hospital, Bad Tolz, Germany; 6 HNO Munchen Nord, Munich, Germany

Correction: *Head Face Med* **18**, 21 (2022)

10.1186/s13005-022-00322-5

Following publication of the original article [1], the author reported that the published version is falsely labelled as a ‘review’. This should be labelled as an ‘original research’.
